# Infections and Syndromes Caused by Campylobacter

**DOI:** 10.3390/microorganisms14061226

**Published:** 2026-05-29

**Authors:** Cecilia Hernández-Cortez, Andres Saldaña-Padilla, Luis Fernando Muñoz-Mateo, Luis Uriel Gonzalez-Avila, Roger Orlando Medina-de-la-Cruz, Graciela Castro-Escarpulli

**Affiliations:** 1 Laboratorio de Bioquímica Microbiana, Departamento de Microbiología, Escuela Nacional de Ciencias Biológicas, Instituto Politécnico Nacional, Mexico City 11340, Mexico; cecihercor6@gmail.com (C.H.-C.); andres950123@hotmail.com (A.S.-P.); 2 Laboratorio de Investigación Clínica y Ambiental, Departamento de Microbiología, Escuela Nacional de Ciencias Biológicas, Instituto Politécnico Nacional, Mexico City 11340, Mexico; louis.qbp@hotmail.com (L.F.M.-M.); u_gza@hotmail.com (L.U.G.-A.); rmc230798@gmail.com (R.O.M.-d.-l.-C.)

**Keywords:** *Campylobacter*, identification, Guillain-Barré syndrome, diarrhoea, antimicrobial resistance

## Abstract

In recent years, diseases caused by species of the genus *Campylobacter* have increased, due to improvements in identification methods, but also because, as part of global travel and trade, these species have spread to countries where no cases had previously been reported. The methodologies for their identification, whether classic through culture media and morphological characteristics, or using molecular biology or even proteomics techniques, play a fundamental role in establishing their diagnosis and providing timely treatment. Likewise, epidemiology will help guide this diagnosis when dealing with poorly defined diarrhoea, as well as the control and prevention of these infections. Similarly, expanding information on the relationship between these species and Guillain-Barré syndrome will lead to a better understanding and timely identification. We must not forget that both intrinsic and acquired antimicrobial resistance are key factors to consider for the successful treatment of infections caused by *Campylobacter* species.

## 1. Introduction

The word *Campylobacter* comes from the Greek terms καμπὐλoς (kampylos) and βακτήρια (bakteria), which mean ‘crooked stick’ and ‘bacteria’, respectively. The latter refers to the characteristic S shape of this bacterium. *Campylobacter* species are the cause of campylobacteriosis, considered the most common source of bacterial gastroenteritis worldwide. In most cases of campylobacteriosis, the disease is mild and self-limiting; however, in young children, the elderly, and immunocompromised individuals, it can be fatal. Members of the *Campylobacter* genus are the leading cause of foodborne bacterial infections, particularly through the consumption of undercooked chicken, contaminated water, or dairy products [1,2,3].

## 2. Campylobacter

The genus *Campylobacter* is part of the *Campylobacteraceae* family, the *Campylobacterales* order and the *Epsilonproteobacteria* class, which includes closely related genera such as *Dehalospirillum*, *Sulfurospirillum* and *Arcobacter*. The genus *Campylobacter* currently consists of 34 officially described species, 11 subspecies and 4 biovarieties (Table 1). *Campylobacter* species are motile, Gram-negative, non-spore-forming bacilli with a spiral-shaped, curved rod shape, measuring 0.5 to 5 μm in length and 0.2 to 0.8 μm in width. Nonetheless, some species have a straight bacillus morphology. Other species, such as *C. gracilis*, *C. hominis*, *C. ureolyticus*, and *C. blaseri*, are non-motile. They are microaerophilic, meaning they grow optimally in atmospheric conditions with 5% to 10% oxygen. All grow at 37 °C; however, some perform better at different temperatures, such as *C. jejuni*, which grows best at 42 °C [1,4,5].

Fundamentally, the gastrointestinal tract of vertebrates such as birds, pigs, sheep, cattle and dogs is its main habitat; however, *Campylobacter* can also be found in mussels. Most are prevalent in varied species of birds, as these animals have a high body temperature. In addition, there are non-zoonotic *Campylobacter* species (*C. concisus*, *C. showae*, *C. gracilis*, *C. ureolyticus*, *C. curvus*, and *C. rectus*) that are found in the microbial community of the human mouth and have the potential to cause periodontitis. Gastroenteritis in humans is caused mainly by the bacterium *C. jejuni* and, to a lesser extent, by *C. upsaliensis*, *C. fetus*, *C. coli* and *C. lari* [1].

*C. coli* and *C. jejuni* are the main zoonotic agents of severe gastroenteritis in humans worldwide, even more so than infections caused by *Salmonella* sp. The infection includes bloody and watery diarrhoea, as well as very painful abdominal cramps [1].

*Campylobacter jejuni* is an important human pathogen, recognised as one of the main causes of bacterial gastroenteritis. Gastroenteritis caused by *C. jejuni* is a zoonotic disease that spreads to humans through animals, especially poultry, which are part of the normal intestinal microbiota, although it can also spread from pigs, cattle and sheep, as well as domestic dogs and cats, according to the latest evidence. *C. jejuni* infection in humans is usually caused by contact with contaminated food from animals, especially poultry, as well as contaminated milk and water [9].

*Campylobacter coli* is a less common human pathogen than *C. jejuni* and appears to be responsible for approximately 10% of *Campylobacter* infections, although higher rates have been reported. In general terms, it is not possible to clinically differentiate between infection caused by *C. jejuni* and *C. coli* [10].

Some other species associated with gastrointestinal infections, but which are not as common, are the following: *Campylobacter lari* is generally found in coastal areas and ocean environments. It is mainly associated with coastal birds, but it is also linked to shellfish and marine mammals. *Campylobacter concisus* colonises the human oral cavity and consists of two genomospecies (GS1 and GS2) that cannot be differentiated in phenotypic terms. Nonetheless, both genomospecies contain numerous strains that have been isolated in patients with diarrhoea and healthy individuals, which makes it difficult to establish their pathogenicity. Specifically, *C. concisus* GS2 appears to be more pathogenic because it is more frequently isolated in clinical patients with bloody diarrhoea. *Campylobacter upsaliensis* has been isolated in clinical cases of bloody diarrhoea worldwide, but it is common in domestic animals such as dogs and cats. This variety of *Campylobacter* is closely related to *C. coli* and *C. jejuni*, according to studies of the *16S* ribosomal gene. *Campylobacter fetus* has the ability to cause severe systemic infections and intestinal disorders. Infections have a greater impact on individuals whose profession exposes them to infected animals, with sheep and cattle being the main reservoirs. Products derived from these animals are presumed to be the cause of infections in humans, although it is rarely isolated in food. Infection with *C. fetus* should be suspected, especially in people with fever of unknown origin who are immunocompromised or who have been exposed to ruminants [11,12].

### 2.1. Virulence Factors

*Campylobacter* virulence consists of several pathways, such as flagellum-mediated motility, the ability to invade, the capacity to generate toxins, and bacterial adhesion to the intestinal mucosa. For *Campylobacter* to successfully colonise the host’s gastrointestinal tract, it needs different virulence factors [4].

#### 2.1.1. Mobility, Chemotaxis, and Stress Response

The amphitrichous and polar flagella of *Campylobacter* are multifunctional, as they participate in mobility, chemotaxis, adhesion to host cells, secretion of virulence elements, autoagglutination, establishment of microcolonies, creation of biofilms, and evasion of the innate immune system. *Campylobacter* flagellin is highly glycosylated, which marks a significant difference from other bacterial flagella, and is composed of two proteins, FlaA and FlaB [13].

Since, unlike other enteric pathogens, *C. jejuni* does not have many adaptive responses, it is sensitive to various environmental stress conditions. Nonetheless, *Campylobacter* has been shown to be able to react adaptively to acidic and aerobic conditions. Exposure to acidic conditions can cause *C. jejuni* to become viable but non-culturable (VBNC). It has different systems that allow it to respond to heat shock, such as RacRS regulation. GroESL is a chaperonin system comprised of two separate proteins (GroES and GroEL), DnaJ, and Lon. Nevertheless, the mechanism of response to oxygen and its products has not yet been elucidated [14].

#### 2.1.2. Phase Variation

Phase variations allow *C. jejuni* to adapt to changes in its environment or to rapidly adapt to alterations in the host system. Several structural genes of *Campylobacter* are subject to phase variation, such as the lipooligosaccharide (LOS) and capsular polysaccharide (CPS) loci, in addition to those encoding flagellar structures [13].

#### 2.1.3. Secretion Systems

The *Campylobacter* type III secretion system (fT3SS) is located in the flagellum core, which secretes various proteins such as Cia, FlaC, and Fed (co-expressed flagellar determinants) for flagellum biosynthesis. The *Campylobacter* invasion antigen (Cia) proteins actually increase the production of pro-inflammatory cytokines during an infection. The type VI secretion system T6SS has been identified in some *Campylobacter* lineages. This system is essential for adaptation to bile acids, which promotes intestinal colonisation [1,15].

#### 2.1.4. Toxins

To date, cytolethal distending toxin (CDT) has been identified and confirmed as the only toxin produced by *Campylobacter*. Several species of the genus, such as *C. jejuni*, *C. lari*, *C. coli*, *C. fetus*, and *C. upsaliensis*, are responsible for its production. When *C. jejuni* is inside the cell, it releases the genotoxin CDT, which causes cell cycle arrest, inflammation and cell swelling. Three genes, *cdtA*, *cdtB*, and *cdtC*, encode the tripartite protein toxin CDT, which belongs to the DNase I protein family [13,14].

### 2.2. Pathogenicity Mechanisms

Although the exact process of infection in humans is still not known with certainty, it is possible to distinguish three fundamental stages. First, the intestine is colonised, especially the crypts of the intestinal mucosa. Next, the proteins of the host epithelium are specifically adhered to. After that, the bacteria invade the intestinal cells and translocate, either paracellularly or transcellularly. *Campylobacter* grows in the intestinal mucosa, and the toxins then cause necrosis of the intestinal villi. The deterioration of the intestinal epithelium triggers a loss of function, the opening of the protective barrier and tight junctions, inflammation, the release of electrolytes from the host’s systemic compartment into the intestinal lumen, and, ultimately, severe bloody diarrhoea. Furthermore, the binding of the bacterium to epithelial cells is accompanied by a potent pro-inflammatory immune response [1].

#### 2.2.1. Adhesion

For *Campylobacter* to colonise, it must adhere to the host’s intestinal epithelium. *C. jejuni* has a variety of different adhesins which, individually or in combination, enable it to adhere to various cellular structures of the host. Presumed adhesins include outer membrane proteins (OMPs), lipopolysaccharide (LPS), and flagella. The three adhesins of *C. jejuni* that have been most thoroughly investigated and confirmed are: CadF (*Campylobacter* adhesion to fibronectin protein), FlpA (a fibronectin-like protein), and JlpA (jejunal lipoprotein A), which bind to heat shock protein 90 in host cells, as well as fibronectin. The CadF protein consists of 319 amino acids and has a molecular mass of 37 kDa. It is encoded by the *cadF* gene, which is conserved and present in almost all isolated *C. jejuni* strains (~95%), as is *flpA*. It is present on the bacterial surface and binds specifically to fibronectin (Fn) [1,13].

#### 2.2.2. Invasion

After adhering, *C. jejuni* penetrates cells, mainly through endocytosis, in a process that requires *Campylobacter*-induced reorganisation of the cytoskeleton via microtubules and microfilaments. Membrane protrusion, facilitated by the small Rho-GTPases Rac1 and Cdc42, is the first step in the invasion process. It is also believed that the flagellum plays a role in invasion through proteins secreted by the T3SS apparatus. Secreted proteins are essential for invasion and colonisation and are introduced into the cytoplasm via the flagellar secretion system. Certain proteins, such as Cia (CiaB, CiaC and CiaI), contribute to effective colonisation and invasion and are also important for intracellular survival [1].

#### 2.2.3. Translocation

*C. jejuni* can migrate from the apical to the basolateral cell surface through translocation. *C. jejuni* has the ability to bind to fibronectin and then invade epithelial cells because it can access the basolateral cell surface. Furthermore, it has been suggested that translocation to underlying tissues is beneficial for *C. jejuni* since it provides access to nutrients such as iron and prevents peristaltic forces in the intestine from eliminating it, making it easier for it to remain there. Several hypotheses describe how *Campylobacter* penetrates the basolateral part of the intestine, crossing tight junctions and the cell barrier, via the transcellular and/or paracellular pathway of *C. jejuni*. First, it enters the apical cell surface by endocytosis and then creates a transient *Campylobacter*-containing vacuole. Through this conduit, the bacteria are released into the lumen or move from the cell to the basolateral side. Alternatively, there is evidence that *C. jejuni* crosses the epithelium via a paracellular pathway, which involves opening tight and adhesive junctions using proteases (such as the serine protease HtrA, which cuts proteins such as occludin and E-cadherin) and then invading the intestinal cells of the epithelium from the basolateral side [1,9].

## 3. Identification Methods for Diagnosis

**Figure 1:**
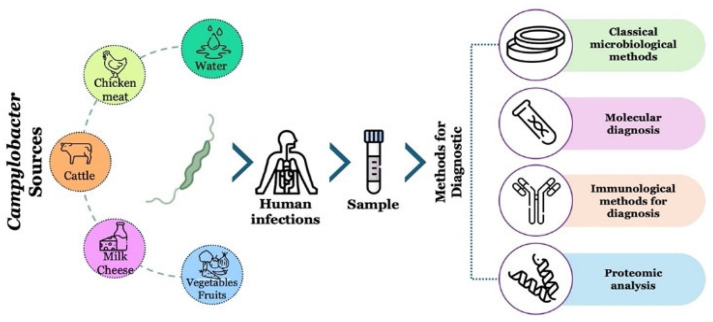
Sources, diseases in humans and diagnostic methods for *Campylobacter jejuni*. This bacterium is isolated from a wide variety of samples and can cause certain diseases in humans. There are various diagnostic methods, with molecular methods being the most effective, followed by immunological methods and, lastly, conventional culture; proteomic analysis, meanwhile, is used for research purposes rather than as a diagnostic method.

The complexity of isolating by conventional microbiological methods and its nutritional requirements increase diagnostic costs. Therefore, different diagnostic methods are currently used for its identification, such as molecular and immunological methods. In addition, the infrastructure required for cultivation such as temperature conditions at 42 °C and a CO_2_ atmosphere (Figure 1) [16].

### 3.1. Microbiological Methods

Initially, the sample to be used for the isolation of *C. jejuni*, which may be faecal matter, is transported in Cary–Blair medium or Amies medium with activated charcoal. The sample can be processed up to 24 h later if stored at 4 °C [16,17].

There are a variety of culture media (Table 2) that allow the isolation of *C. jejuni*, some of which can be supplemented with sheep blood or activated charcoal. Some of the media are considered selective due to the antimicrobial or antifungal components that are added. It is also advisable to supplement them with pyruvate, a component that protects against reactive oxygen species that affect *C. jejuni*, as a nutritional function, and improves tolerance to oxidative stress when the CO_2_ atmosphere is insufficient. Overall, these media are incubated at 37 °C or preferably at 42 °C, since at this temperature only *C. jejuni* grows and other enteropathogens are inhibited, increasing the culture specificity. The time sometimes reaches up to 72 h because this bacterium has relatively slow growth, and incubation under microaerophilic conditions is necessary [18,19].

Due to the difficulty of obtaining selective media in the laboratory, alternative microbiological methods have been proposed. The passive filtration method proposed by Steele and McDermott in 1984 allows *C. jejuni* to be isolated without the need for selective media. This method requires mixing the faecal sample with 1/10 Phosphate-Buffered Saline (PBS). A 0.45 or 0.65 µm filter is placed on a blood culture medium (non-selective), 100 µL of the suspension is added to the filter, allowing the bacterial cells to disperse on the filter, it is left to stand for 30–45 min at 37 °C, and the filter is removed and incubated at 42 °C in CO_2_. Actually, the microbiological classic method is used for the isolation and identification for diagnosis of *C. jejuni* and other species in human infections because it is the most sensitive method, despite the difficulty of isolating this bacterium, and it is the most common method available in diagnosis centres such as hospitals. Other methods should be compared to establish sensitivity and effectiveness in the identification of *Campylobacter* compared versus culture [23].

After isolating *C. jejuni*, a Gram staining technique is used to examine the characteristic colony morphology, which is described as light-coloured, grey or white punctiform colonies. Under a microscope, Gram-negative bacilli with a curved, thin, spiral or ‘S’ shape are expected to be observed. Occasionally, the polar flagellum can be visible. Identification is also complemented by microbial metabolism tests (Table 3).

*C. jejuni* strains can be rapidly identified and confirmed by agglutination tests using latex particles coated with polyclonal surface antibodies against *C. jejuni*, which detect outer membrane proteins or flagella. These tests are currently commercially available and are notable for their speed and high specificity. Under this same concept, the virulence of the strain can be corroborated by an in vitro autoagglutination test. For this procedure, cells are collected from a culture medium and placed in PBS pH 7.2. The suspension is adjusted to an optical density (A_600_) of 1.0 to 1.5, and a 2 mL portion is allowed to sediment at 25 °C for 24 h, then a portion of the surface is carefully taken, and the optical density (A_600_) is measured again. If it decreases, it means that the bacteria have sedimented due to autoagglutination [18,26,27,28].

### 3.2. Molecular Methods

Due to the difficulty of isolating *C. jejuni* in a conventional culture, alternative techniques such as molecular methods have been used. The method that is relatively easy to apply, despite its limited availability to most laboratories, is MALDI-TOF (Matrix-Assisted Laser Desorption/Ionization—Time of Flight), which, in the case of *C. jejuni*, allows for correct identification at the genus and species level. The data on the use of MALDI-TOF as a method for identifying *Campylobacter* species indicate a 100% identification rate compared with the reference method for all *Campylobacter* species, except in the case of *C. jejuni*, where the rate is 99.4%, which is relatively lower than that for other species. Conversely, the accuracy of conventional methods compared with MALDI-TOF ranged from 0% to 100% depending on the species. Some authors recommended the use of the MALDI-TOF method to identify *Campylobacter*, for the 0.4% of discrepancy, whereas conventional methods led to 4.5% of discrepancy. Multiple authors refer to the effectiveness of this method with *C. jejuni* and other species, despite the fact that culture remains a limitation, as it requires the isolated strain. Direct detection of bacteria in blood and faecal samples using MALDI-TOF has been reported, which may include *C. jejuni*, omitting plate culture. Techniques using DNA probes for identification are also efficient. The detection of *C. jejuni*-specific genes by PCR has also been reported. The genes detected are diverse; the *16S rRNA* gene can be amplified, sequenced, and subjected to bioinformatics analysis. In contrast, genes such as the *hipO* gene, which codes for the enzyme hippuricase (hippurate hydrolase), and the *mapA* gene can be detected. Similarly, the *flaA*, *cadF* and invasion-associated (*iam*) genes, the *cdtABC* operon associated with the cytolethal distending toxin, and the *virB11* and *wlaN* genes present in plasmids can also be detected, and their detection can be used to determine whether the strain is virulent. It should be noted that the only requirement for this molecular test to be specific is that the gene be unique to *C. jejuni*, which is complicated by the phylogenetic proximity to other *Campylobacter* species, as well as other genera such as *Aliarcobacter* [29,30,31,32,33,34].

It is difficult to determine the effectiveness of molecular methods, as few centres use them as a routine diagnostic tool; however, they offer a higher detection rate. For example, conventional PCR or qPCR has been shown to be more sensitive in detecting *Campylobacter*—up to 97%—compared with culture; it is a rapid method that provides results within a few hours; its main advantage is the detection of viable but non-culturable cells, although it does not distinguish between live and dead cells; DNA extraction methods can be ineffective and require a qualified analyst [29,30,31,32,33].

### 3.3. Immunological Methods

Other tests currently available, which are also simple, rapid, and specific, are those based on immunochromatography, whose positive result can be interpreted by the appearance of a coloured reaction caused by the antigen-antibody interaction on nitrocellulose strips. These are generally commercial methods. These tests allow for an accurate and rapid diagnosis of infections caused by *C. jejuni*, as well as enabling timely treatment if the doctor considers it necessary to act quickly. Some of these tests are capable of detecting *C. jejuni* antigens directly in a faecal sample [35].

Immunological methods that detect IgM or IgG antibodies in serum are effective. This requires sending blood samples to the laboratory for testing. The main advantage of these methods is that antibodies can be quantified, allowing the progress of the infection to be monitored. This is particularly important in the diagnosis of syndromes such as Guillain-Barré syndrome. These studies can be complemented by a sandwich ELISA (Enzyme–Linked Immunosorbent Assay) or indirect ELISA method, in which antibodies, specifically interleukin-8 (IL-8), are detected even in a faecal sample or directly in blood. Although these tests are sensitive up to a 96% because they detect low amounts of bacteria and are highly specific with 99% in comparison with culture, it is necessary to consider that some factors reduce their success, due to the use of antibiotics prior to analysis, generating false negatives. Finally, these techniques show similarity in diagnosis compared to molecular methods [35,36].

### 3.4. Proteomic Methods

Proteomic analysis of *C. jejuni* strains offers a comprehensive approach to understanding the complete biology of this bacterium. Although it is not a method of identification, it provides information on the physiology and biochemical mechanisms of the bacterium’s metabolism, the existence of pathogenicity mechanisms reflected in the virulence of the strains, and possible mechanisms of resistance to antimicrobials and other toxic agents that the bacterium may encounter. This type of study may include Liquid Chromatography-Tandem Mass Spectrometry methods (LC–MS/MS), the mass spectrometry-based phyloproteomics (MSPP), the Sawn-Off or Shotgun Proteomic Analysis (SOSPA) method and the isotopic labelling technique (SILAC/iTRAQ). The proteomic methods are used to research, but the availability for clinical diagnosis is limited. These tools offer an opportunity to understand the molecular context of *Campylobacter* and other bacteria and the diversity of pathogenicity mechanisms, antimicrobial resistance and metabolic pathways [37,38,39,40,41,42].

## 4. Source and Transmission

The genus *Campylobacter* is of global clinical and epidemiological importance due to its role in zoonotic diseases transmitted to humans. Despite being commensal organisms in many warm-blooded animals, including poultry, cattle, pigs and sheep, wild birds and pets such as dogs and cats, the main etiological agents associated with disease in humans are *C. jejuni* and *C. coli*, which trigger infections in humans [43,44,45].

### 4.1. Animal and Environmental Reservoirs

The main reservoirs of *Campylobacter* are food-producing animals, especially poultry (chickens), in whose intestine the bacterium can be found at high levels without causing clinical disease in the host. Meat contamination arises during slaughter, as it usually occurs through contact with infected faeces, making raw or undercooked meat products one of the most important sources for human infection [46].

However, this is not the only source; additionally, *Campylobacter* can be found in untreated water (wells, streams) or in ice, both contaminated by the faeces of infected animals, as well as in unpasteurised dairy products. The bacteria do not multiply significantly outside the host, but their infectious dose is exceptionally low; consequently, small amounts in food or water can cause an infection [47,48,49].

### 4.2. Routes of Transmission

Transmission in humans is predominantly faecal-oral, occurring through:

- Direct Ingestion: Consumption of undercooked or contaminated poultry meat; considered the main source of sporadic cases, as well as through the ingestion of raw milk or contaminated water [46,50].
- Cross-Contamination: A critical point in the home or restaurants during food processing and preparation, when utensils, cutting boards, hands, or surfaces come into contact with contaminated raw products and are not properly sanitised, allowing microorganisms to be subsequently transferred to ready-to-eat foods, which increases the risk of gastrointestinal infections [46,50].
- Direct Contact: Transmission can also occur through handling infected animals or their faeces and, to a lesser extent, via person-to-person transmission, especially in close care settings. Likewise, recreational activities in contaminated waters have been documented as a cause of infection [46,51,52].

The global distribution of *Campylobacter* and the diversity of sources make the exact quantification of the contribution of each transmission route to the total burden of human disease difficult, but strategies that reduce prevalence in live birds have been shown to decrease human cases of campylobacteriosis in certain countries [53,54].

## 5. *Campylobacter* Infections

*Campylobacter* infections encompass a clinical spectrum ranging from self-limiting gastrointestinal conditions to systemic manifestations and post-infectious complications of variable severity. The disease caused by *Campylobacter* is called campylobacteriosis and constitutes one of the main causes of bacterial gastroenteritis worldwide, surpassing *Salmonella* and *Shigella* in incidence in many countries [53,54,55,56,57].

The species most frequently implicated in human pathology are *C. jejuni* (responsible for approximately 80–90% of cases) and *C. coli* [57,58].

### 5.1. Gastrointestinal Infections

The most frequent clinical presentation of *Campylobacter* infection is gastroenteritis. After a usual incubation period of 2 to 5 days following the ingestion of contaminated food or water, patients develop symptoms that include [47,57]:

- Diarrhoea, initially watery, which in a significant percentage of cases may become bloody.
- Abdominal pain and cramps (colic), often intense, which can mimic an acute abdomen.
- Fever and general malaise, accompanied in some patients by nausea and vomiting.

These symptoms are usually self-limiting, resolving in most cases around 5 to 7 days after onset, mostly without the need for antibiotic treatment. The condition can occur in healthy people of any age, but groups at higher risk of severe disease or complications include young children, the elderly, and people with compromised immune systems. In these groups, the infection may present with longer duration, higher bacterial load, and risk of systemic dissemination [58,59].

#### Post-Infectious Complications

Although *Campylobacter* gastroenteritis is usually mild and self-limiting, both acute and late complications have been described, among which the following stand out:

- Bacteraemia: Invasion of the bloodstream is infrequent but can occur, especially in immunocompromised patients. It is associated with persistent fever and risk of sepsis.
- Reactive Arthritis: Can appear days or weeks after the gastrointestinal episode, characterised by joint pain and inflammation, with a predilection for knees and ankles. Its pathogenesis is related to autoimmune mechanisms triggered by bacterial antigens [60].

### 5.2. Cancer

The relationship between bacterial infections and cancer has gained increasing attention, particularly in the context of the gut microbiota. While several bacterial species have been implicated in carcinogenesis, evidence supporting *Campylobacter* as a direct human carcinogen remains limited and inconclusive [61].

Epidemiological data does not support a strong or consistent association between *C. jejuni* infection and overall cancer risk. For instance, a large-scale study with a 10-year follow-up did not detect a significant global increase in cancer incidence after infection, although some associations with specific cancer types in subgroups were reported, suggesting that any potential relationship is likely context-dependent rather than generalisable [62].

From a mechanistic perspective, certain *Campylobacter* strains produce cytolethal distending toxin (CDT), a well-characterised genotoxin involved in bacterial pathogenesis. CDT has been experimentally shown to induce DNA damage in host cells through the DNase-like activity of its CdtB subunit, leading to double-strand breaks and activation of the DNA damage response, including pathways involving ATM, ATR, and p53. These events can result in cell cycle arrest at the G2/M phase, apoptosis, or cellular senescence. Importantly, in cases of incomplete DNA repair, CDT exposure may contribute to genomic instability [61,63,64,65,66].

Nevertheless, it is important to emphasise that most of this evidence derives from in vitro or experimental models, and a direct causal link between CDT activity and cancer development in humans has not been established. Therefore, while CDT-mediated genotoxicity represents a plausible biological mechanism, its role in carcinogenesis remains hypothetical. In the broader context of the gut microbiome, chronic inflammation and dysbiosis are recognised contributors to colorectal carcinogenesis. Although this relationship is well supported for microorganisms such as *Fusobacterium nucleatum* and colibactin-producing *Escherichia coli*, the contribution of *Campylobacter* is less well defined and has been proposed only as a potential cofactor [67,68].

In summary, current evidence supports the genotoxic potential of *Campylobacter* through CDT production; however, there is no definitive evidence establishing this genus as a direct carcinogenic agent in humans. Further studies are required to clarify whether *Campylobacter* plays a contributory role in cancer development under specific biological or environmental conditions.

### 5.3. Guillain-Barré Syndrome

Guillain-Barré syndrome (GBS) is considered an autoimmune disorder of the peripheral nervous system, characterised by acute or subacute symmetrical ascending motor weakness, dysreflexia, and mild to moderate sensory abnormalities. This syndrome was partially described in 1859 by Landry as an ‘ascending paralysis,’ but it was definitively characterised as a distinct nosological entity in 1916 by Guillain, Barré, and Strohl, who described its clinical presentation, the biochemical characteristic of albumin cytological dissociation, and electrophysiological findings, thus distinguishing it from other infectious neuropathies [69,70].

**Figure 2:**
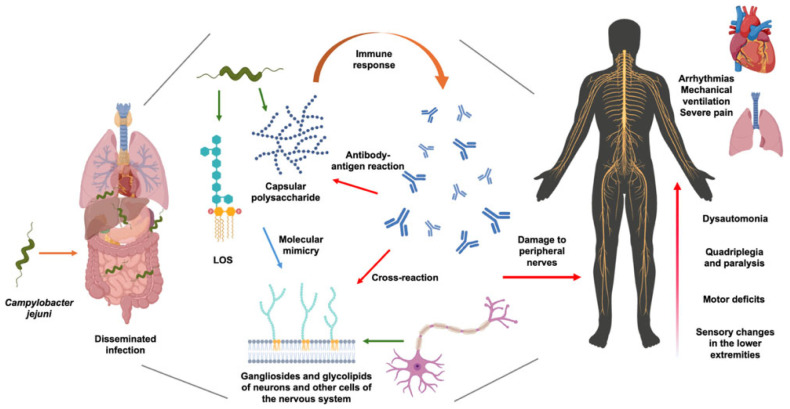
Pathogenesis of Guillain-Barré syndrome. *Campylobacter* infection triggers the production of antibodies against lipooligosaccharides (LOS), amongst other antigens. Due to the presence of certain molecules in the *Campylobacter* membrane that are very similar to molecules in the host cell membrane, including LOS, a cross-reactive immune response may occur primarily affecting the neurons of the peripheral nerves and, over time, the rest of the nervous system.

It has been reported that, in the weeks leading up to the onset of GBS, 60% of patients experience a gastrointestinal or respiratory infection. In between 26% and 41% of cases, the pathogen most associated with the syndrome is *Campylobacter jejuni*. The pathogenic mechanism of the disease involves the immune system mistakenly identifying its own nerve cells as parts of the bacterium and attacking them, because lipooligosaccharides (LOS) closely resemble the gangliosides in nerves, triggering an autoimmune response that damages the myelin and nerve axons. The antibodies generated against *C. jejuni* cross-react with antigens in the peripheral nerve axons and myelin; serum IgM and IgG antibodies against gangliosides or glycolipid complexes are detected in up to 92% of patients with the syndrome during the acute and active phase of the disease, with their levels decreasing in the following weeks (Figure 2). The gene encoding Cst-II (sialyltransferase) in *C. jejuni*, which is necessary for the biosynthesis of LOS structures similar to GM1 and GD1 gangliosides, is the bacterial marker that has been correlated with SGB [69,70,71,72,73,74].

The immune response to SGB-associated bacteria varies from person to person; therefore, other reasons why one might contract this disease include: genetic differences in the immune system and the high variability of the surface antigens possessed by *C. jejuni*, due to the relatively large number of hypermutable simple sequence repeat (SSR) regions in the bacterium’s genome, as the presence of multiple SSR-mediated variable-phase genes encoding enzymes that modify surface structures, including the capsular polysaccharide (CPS) and LOS, creates extreme diversity in the cell surface within bacterial populations and thus promotes adaptation to selective pressures in host environments [74].

The syndrome is classified into three main subtypes according to electrophysiological findings, namely acute inflammatory demyelinating polyneuropathy, acute motor axonal neuropathy, and acute sensory-motor axonal neuropathy. However, other references indicate that there are four basic patterns, which also include Miller-Fisher syndrome. Typically, GBS progresses through three distinct phases: progression, plateau and recovery. GBS begins with muscle weakness that progresses rapidly within a few days; it has been reported that in 30% of patients, this can lead to respiratory failure requiring mechanical ventilation and admission to intensive care units. Sensory symptoms (pain and paraesthesia) and autonomic dysfunction also occur, which can result in life-threatening haemodynamic instability or arrhythmias. Most deaths caused by this syndrome occur within the first 6 months after onset, with the risk of mortality remaining elevated for several years beyond the acute phase of the disease; even in patients who achieve functional recovery after this phase, prolonged pain and fatigue may still be experienced, with reports indicating that the prevalence of residual pain and other sensory deficits varies between 33% and 82%, depending on the location of the symptoms, the muscle group affected in those with chronic residual muscle pain, and the subtype of sensory deficit; such as paraesthesia, dysaesthesia, radicular or neuropathic pain, and allodynia, all of which are associated with impaired quality of life. Pain intensity correlates with disability, loss of strength and fatigue [70,71,73,74].

The heterogeneous clinical presentation and progression of symptoms in GBS can lead to a delay in diagnosis during the active phase of the disease, requiring treatment that rapidly and completely inhibits the key pathophysiological components of the disease once the diagnosis has been confirmed. Diagnosis is based primarily on the patient’s medical history and clinical assessment, with additional tests such as lumbar puncture and electroneuromyography used to confirm and differentiate between subtypes [70,71].

## 6. Epidemiology

According to the World Health Organisation (WHO), diarrhoeal diseases caused by consuming contaminated food affect 550 million people annually, including 220 million children under the age of five. *Campylobacter* spp. is one of the main causes of childhood diarrhoea worldwide. Every year, almost 1 in 10 people fall ill. In developing countries, infections caused by this microorganism in children under the age of 2 are particularly common and can sometimes be fatal. Consequently, it has been reported that the high incidence of *Campylobacter* diarrhoea, its duration and potential complications make it a problem of significant socioeconomic importance. Updated estimates, with projections up to January 2026, identify *Campylobacter* as the second leading cause of diarrhoea-related morbidity globally, with 291.4 million cases reported annually [58].

In the European Union (EU), campylobacteriosis is one of the most common foodborne illnesses, with over 246,000 cases per year, although the actual figure is thought to be closer to 9 million annually. The EFSA (European Food Safety Authority) estimates that the cost of this disease to public health systems and in terms of lost productivity stands at around €2.4 billion a year. In its assessments, the EFSA found that chickens and chicken meat are the most common sources of infection, accounting directly for between 20–30% of cases in humans [75].

In their 2024 report, the EFSA and the ECDC (European Centre for Disease Prevention and Control) reported that 26 EU Member States notified 168,396 confirmed cases of human campylobacteriosis, corresponding to 55.3 cases per 100,000 inhabitants, representing an increase of 11.9% compared to the figures reported in 2023 (49.4 cases per 100,000 inhabitants) [76].

The CDC estimates that 1.5 million people in the United States fall ill each year due to *Campylobacter*. In the United States, there are an estimated 124 deaths annually, accounting for 6.4% of global deaths from diarrhoea. In 2023, 11,926 cases of *Campylobacter* infection, 2482 hospitalisations and 49 deaths were reported; however, in 2024, only 4312 cases of infection with this microorganism were reported, 929 hospitalisations and 27 deaths. This was due to new reporting requirements for the pathogen, as previously, FoodNet collected data on *Campylobacter* infections diagnosed by culture only, culture-independent diagnostic tests confirmed by culture, or culture-independent diagnostic tests alone. From 2024 onwards, FoodNet no longer collects data on *Campylobacter* infections diagnosed solely by culture-independent diagnostic tests [77].

It has been reported that in temperate regions, there is an increase in cases during the warmer months. The seasonal pattern strongly suggests a causal relationship with increased consumption of undercooked foods and compromised cold chain integrity. Nevertheless, host gene expression may play a role in this marked seasonal pattern [78].

Chickens are considered a natural reservoir for this microorganism, particularly *C. jejuni*, as they provide an ideal biological habitat for its survival and growth due to their high body temperature, especially in their intestinal system. Chickens are usually colonised at two to three weeks of age and are asymptomatic after colonisation. Humans contract infections mainly by consuming animal-derived foods contaminated with the microorganism, particularly raw or undercooked poultry, untreated water, unpasteurised milk and dairy products, with campylobacteriosis in humans being the most common cause of foodborne illness worldwide. *Campylobacter* spp. usually causes less severe diarrhoea but can cause serious chronic or systemic infections in young children, the elderly, and immunocompromised individuals. The spread of infection beyond the gastrointestinal tract can have serious consequences, such as cholecystitis, pancreatitis, peritonitis, or significant gastrointestinal bleeding. Although rare, manifestations such as meningitis, endocarditis, septic arthritis, osteomyelitis, and neonatal sepsis have been reported. *Campylobacter* spp. infections are associated with various complications, such as the non-paralytic form of Guillain-Barré syndrome (GBS), Miller-Fisher syndrome, and reactive arthritis [79].

Guillain-Barré syndrome (GBS) is a neuromuscular emergency and the most common and severe acute paralytic neuropathy worldwide. It is the most common cause of acute flaccid paralysis, with an approximate annual incidence of 0.3–0.6 to 4–6.08 cases per 100,000 inhabitants worldwide, depending on geography, age, exposure to infections, and other risk factors, with high incidences reported in low- and middle-income countries. The incidence is slightly higher in men than in women and increases with age [69,70,71,73,74].

Reports of GBS have been based mainly on studies carried out in Europe and North America. However, significant outbreaks of this syndrome have been observed in developing Latin American countries such as Peru and Colombia [74].

In 2023, Peru’s National Centre for Epidemiology, Disease Prevention and Control reported an unusual increase in the number of GBS cases compared with previous years, declaring a national health emergency that year, as between epidemiological weeks 1 and 28, a total of 231 suspected cases of GBS were reported in 20 of Peru’s 24 departments. In 2025, 183 cumulative cases were recorded, and so far in 2026, 120 cases have been recorded [80,81].

In Colombia, a total of 3122 cases of GBS were reported between 2016 and July 2023; and between 2019 and 2022, an average of 251 cases per year was recorded, indicating that there has been no unusual increase in cases in that country, unlike in Peru. In this country, cases of GBS are reported as part of the surveillance of Acute Flaccid Paralysis (AFP) in children under 15 years of age, and so far in 2026, 81 cases have been recorded [74,82].

In Mexico, GBS is also classified as a case of AFP in children under 15 years old, and reporting is mandatory. In 2024, the Ministry of Health reported that, up to epidemiological week 12, 81 cases of AFP had been reported across 30 municipalities in the state of Tlaxcala, of which 42 samples tested positive for *C. jejuni*, with 34 cases classified as GBS and four deaths recorded. In 2024, 133 cumulative cases were recorded, and up to epidemiological week 15 of 2026, there have been 115 recorded cases of AFP. It is also worth noting that in Mexico, there is no routine or targeted screening for *Campylobacter* in cases of gastrointestinal infections; consequently, it is classified under “Intestinal infections caused by other organisms” and “Poorly defined infections”, with a cumulative total of 991,903 cases recorded in 2025 and 1,018,884 cases recorded up to epidemiological week 15 of 2026, where it is likely that in some of these cases the causative agent of these infections is *Campylobacter* [83].

Regarding Asia, an outbreak of Guillain-Barré syndrome was recently reported in Pune (India) where, as of March 2025, the total number of cases was 225, with 197 confirmed diagnoses and 28 suspected cases, considered one of the largest outbreaks in that city. Eleven deaths from this syndrome were also reported. One of the microorganisms involved was found to be *C. jejuni* (25 positive samples from patients with GBS), highlighting how cases of infection by this bacterium are increasing rapidly due to the water shortage in that area. It is therefore necessary to understand the importance of maintaining solid sanitation infrastructure, public education on hygiene practices, continuous surveillance and an understanding of the pathogenesis of GBS, among other things, in order to minimise the risk of future outbreaks of this disease [84].

## 7. Treatment

Most enteric *Campylobacter* infections are generally self-limiting, and palliative care is typically recommended. Treatment is primarily based on oral or parenteral rehydration; furthermore, it is important to avoid the administration of agents that restrict gastrointestinal transit to prevent the prolongation of symptoms. Nevertheless, in immunocompromised patients with prolonged infections or those who develop a systemic process, antimicrobial therapy may be required [85,86,87].

In recent years, researchers have studied the use of natural compounds such as polyphenols (flavonoids, stilbenes, phenolic acids) as alternatives to antibiotics or as adjuncts to antibiotic therapy. In addition to biological activities, these compounds possess antioxidant, anti-inflammatory, and neuroprotective properties, among others, which classify them as compounds with significant health benefits. These compounds may help control campylobacteriosis at different stages through various mechanisms, such as regulating gene expression, inactivating enzymes, and altering the membrane; however, the applicability and safety of these compounds are still under investigation [88].

In cases where antibiotic intervention is required (dysenteric symptoms, high fever, severe abdominal pain), the currently recommended treatment consists of macrolides, particularly as azithromycin or erythromycin; this treatment is commonly sufficient to eliminate the infection. Ciprofloxacin is a fluoroquinolone commonly used to treat enteric infections; however, the reason macrolides have been chosen as the therapeutic agents of choice is due to the increasing rates of resistance to ciprofloxacin, not only in clinical isolates but also in food isolates [79,89].

## 8. Antimicrobial Resistance

Infections caused by *Campylobacter* species have had an impact on public health due to the high incidence of multidrug-resistant strains in recent decades. It is well-described that severe cases of Campylobacteriosis can be easily treated with first-line antimicrobials for enteric infections, such as azithromycin, erythromycin, clarithromycin, tetracycline, and ciprofloxacin; nevertheless, various studies have demonstrated that *Campylobacter* spp. may possess intrinsic and/or acquired factors that promote antibiotic resistance, primarily to macrolides and fluoroquinolones. The high prevalence of *Campylobacter* species in the poultry industry leads to the use of antimicrobials in water or feed to control the disease; however, it is precisely this use of antimicrobials that leads to the spread of residues that reach both consumers and other environments, so the increase in antibiotic resistance is mainly linked to their excessive use in the poultry and animal-derived food production industries, as well as the incorrect use of antibiotics to treat certain diseases [86,88,89,90,91,92].

**Figure 3:**
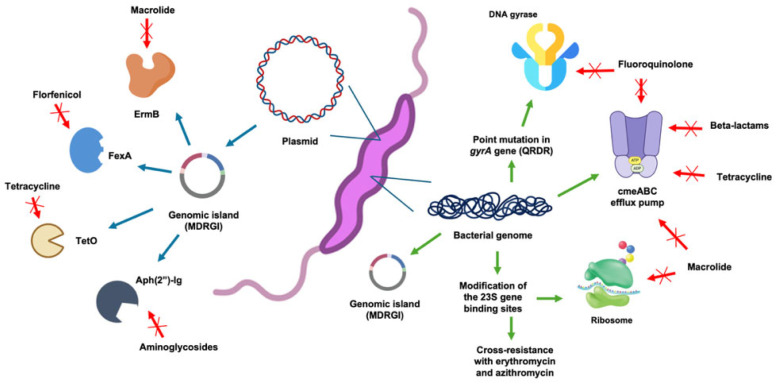
Mechanisms of antimicrobial resistance in *Campylobacter* spp. antimicrobial resistance may be encoded in plasmids or in the chromosome; in the latter case, it may be due to mutations or genes encoding efflux pumps. MDRGI: Multidrug resistance genomic island.

The mechanisms and genetic determinants leading to resistance to different antibiotic families have been detected primarily in the bacterial genome as well as in mobile genetic elements (plasmids), especially those conferring resistance to aminoglycosides (Figure 3) [90,93].

In Taiwan, 219 human isolates (40 of *C. coli* and 179 of *C. jejuni*) were studied between 2016 and 2019. This study demonstrated that a large proportion of the isolates (100% of *C. coli* and 88.3% of *C. jejuni*) were multidrug-resistant. Furthermore, the presence of resistance genes associated with horizontal transfer, such as *erm(B), tet*, and *blaOXA*, among others, was detected. A total of 62.5% of the *C. coli* isolates were resistant to azithromycin, clindamycin, and erythromycin, which complicates the treatment of infections in this region of Asia [94].

A study published in 2024 reports the presence of 69 *C. jejuni* isolates in various samples from both poultry meat and farm workers. Worryingly, 86% of these isolates exhibited resistance to more than one antibiotic, predominantly ciprofloxacin, erythromycin, tetracycline, and azithromycin, among others. This demonstrates the importance of studying MDR *C. jejuni* and its negative impact on public health [34].

A retrospective study conducted in 2025 demonstrated that, in European and Asian countries, *C. jejuni* and *C. coli* present fluoroquinolone resistance rates between 20 and 96.5% in clinical isolates; in the case of macrolides and tetracyclines, resistance rates are high, mainly in the United States, Portugal, and Spain. The main mechanisms of resistance to the antibiotics most commonly used to treat *Campylobacter* infections are described below [90].

### 8.1. Fluoroquinolone Resistance

Fluoroquinolone acts directly on two important enzymes related to DNA synthesis: DNA gyrase and Topoisomerase IV. Various analyses have elucidated that the resistance of *Campylobacter* spp. to this family of antibiotics is driven by point mutations in the quinolone resistance-determining region (QRDR) of the *gyrA* gene. There are different modifications related to the increase in quinolone resistance; likewise, the most frequent amino acid modification is the C257T change in the *gyrA* gene, which produces the Thr86Ile substitution and confers a high level of resistance. Another mechanism by which *C. jejuni* acquires resistance to fluoroquinolones involves the cmeABC efflux pump [95,96].

### 8.2. Macrolide Resistance

Macrolides are antibiotics with a broad spectrum of action against Gram-positive and Gram-negative bacteria, including *Campylobacter*, which is why they are widely chosen for the treatment of multiple infections. Resistance rates to erythromycin in clinical, animal, and food isolates have remained around 10%; beyond this, in recent years, the resistance rate in *Campylobacter* isolates has increased, particularly in *C. coli* [90,97].

The activity of macrolides is based on the interruption of protein synthesis upon binding to the ribosome. Resistance results from a modification in the binding sites of the 23S rRNA gene, specifically at nucleotides 2074 and 2075; this leads to high-level resistance (MIC > 512 μg/mL). It is important to clarify that in the *Campylobacter* genome, there are three copies of the *23S rRNA* gene, and it is possible that mutations are not present in all three copies, which decreases macrolide resistance levels. Cross-resistance between erythromycin and azithromycin has been reported when these mutations are present [97,98].

As in the case of fluoroquinolones, various studies demonstrate that the inactivation of the CmeABC efflux pump leads to a decrease in erythromycin resistance levels [98,99,100].

For a while, it was thought that the modification of bases in ribosomal genes was the only mechanism for macrolide resistance in *Campylobacter* species; however, in 2014, the presence of a methylase enzyme, ErmB, was detected, which is responsible for decreasing the binding of the antibiotic to the target site [90,93].

It is important to emphasise that there is a large body of research demonstrating that the *ermB* gene is located within a multidrug resistance genomic island (MDRGI), which can be situated into the chromosome or in plasmids [90].

In addition to this gene, other genes are located within the MDRGI, such as *fexA*, which encodes for florfenicol resistance; *tetO*, for tetracycline resistance; and *aph(2”)-lg*, encoding resistance to aminoglycosides, among other genes; these have been detected in both human and animal *C. coli* and *C. jejuni* isolates [98,101].

### 8.3. Resistance to Other Antimicrobials

*Campylobacter* spp. possesses different mechanisms that confer resistance to a wide range of antibiotics. Beta-lactam resistance is mediated by the activity of beta-lactamase enzymes (penicillinase, OXA-61), the decrease in membrane permeability through a major outer membrane protein (MOMP), and the participation of the CmeABC efflux pump [89].

Tetracycline resistance is related to target site modification by the binding of TetO as well as the activity of the CmeABC efflux pump; in this case, the relationship with decreased membrane permeability has not been well described [95,102].

Antimicrobial resistance in *Campylobacter* spp. constitutes a growing public health problem, as it is with other microorganisms. *Campylobacter* is a commensal microorganism in many animal species; it is exposed to different antibiotics used in many treatments within the veterinary and food production industries. In addition to the overuse of antibiotics in clinical settings, these scenarios are ideal for increasing resistance rates to different groups of antibiotics due to selection pressure, primarily to fluoroquinolones and aminoglycosides.

## 9. Conclusions

Although only two species of *Campylobacter* (*C. jejuni* and *C. coli*) are the main pathogens, other species that are not as frequent in humans but that may emerge as new pathogens should not be ruled out. Due to the great variety of species, the identification of *C. jejuni* is complex using classical microbiological methods; identification can be replaced with other methods, or new molecular techniques can be implemented.

Transmission to humans is multifactorial, occurring via the faecal-oral route, facilitated by an exceptionally low infectious dose that allows infection through direct ingestion, cross-contamination, and contact with the environment.

Cases of campylobacteriosis present as self-limiting gastrointestinal illnesses, but their impact extends beyond this, as they carry risks of post-infectious complications, such as reactive arthritis and bacteraemia, in vulnerable groups. Experimental and epidemiological data suggest a possible indirect contribution of *Campylobacter* in tumour processes. In contrast, some people develop Guillain-Barré syndrome and others do not; it has been hypothesised that the immune response differs among individuals infected with *Campylobacter*. Despite this, the evidence regarding the development of this syndrome lies in the molecular mimicry between bacterial lipooligosaccharides and gangliosides present in peripheral nerves, which triggers an autoimmune response.

In recent years, the rate of antimicrobial resistance in *Campylobacter* has increased for fluoroquinolones and macrolides.

## Figures and Tables

**Table 1:** Species and subspecies of *Campylobacter*.

Species	Subspecies	Species	Subspecies
*C. armoricus*		*C. insulaenigrae*	
*C. avium*		*C. jejuni*	*jejuni* *doylei*
*C. blaseri*			
*C. canadensis*		*C. lanienae*	
*C. coli*		*C. lari*	*lari* *concheus*
*C. concisus*			
*C. corcagiensis*		*C. mucosalis*	
*C. cuniculorum*		*C. novaezeelandie*	
*C. curvus*		*C. ornithocola*	
*C. fetus*	*fetus* *venerealis* *testudinum*	*C. peloridis*	
		*C. pinnipediorum*	*pinnipediorum* *caledonicus*
*C. geochelonis*		*C. rectus*	
*C. gracilis*		*C. showae*	
*C. helveticus*		*C. sputorum*	
*C. hepaticus*		*C. subantarcticus*	
*C. hominis*		*C. troglodytis*	
*C. hyointestinalis*	*hyointestinalis* *lawsonii*	*C. upsaliensis*	
		*C. ureolyticus*	
*C. iguaniorum*		*C. volucris*	

Note: These are the species and subspecies reported in the literature; although, in recent years, new species such as *C. devanensis* have been proposed, which few authors mention but which already appear in the LPSN [6,7,8].

**Table 2:** Culture media for *C. jejuni* isolation.

Media	Added Blood	Antimicrobial Agent	Special Supplement
CCDA (Charcoal, cefoperazone and deoxycholate)	No	Cefoperazone Amphotericin B *	Activated charcoal Ferrous sulphate Sodium pyruvate
Skirrow	5% sheep blood	Polymyxin B Trimethoprim Vancomycin	No
Preston	5% blood (horse or sheep)	Polymyxin B Trimethoprim Rifampicin Cycloheximide *	No
Campy-BAP	5% sheep blood	Vancomycin Polymyxin B Trimethoprim Cephalothin/Cefoperazone Amphotericin B *	*Brucella* medium base
Campy-CVA	5–10% sheep blood	Cefoperazone Vancomycin Amphotericin B *	Blood agar base
Butzler	5–10% sheep blood	Vancomycin Cefoperazone Polymyxin B Trimethoprim	No
Campy-Cefex	5% sheep blood	Cefoperazone Cycloheximide *	Activated charcoal Can use horse blood

Refs. [20,21,22] * antifungal.

**Table 3:** Biochemical tests for the identification and differentiation of *Campylobacter* spp.

Test	*C. jejuni*	*C. coli*	*C. fetus*
Oxidase	+	+	+
Catalase	+	+	+
Nitrate reduction	+	+	+
Urease	–	–	–
Hippurate hydrolysis	+	–	–
Growth at:			
25 °C	–	–	+
37 °C	+	+	+
42 °C	+	+	–

Refs. [24,25]. + positive, − negative.

## Data Availability

The original contributions presented in this study are included in the article. Further inquiries can be directed to the corresponding author.
